# Applicability of Hf-Free 247LC as a Filler Metal for Hot Crack-Free 247LC Superalloy Welds Assisted by Varestraint Testing

**DOI:** 10.3390/ma18061284

**Published:** 2025-03-14

**Authors:** Seong-Jin Lee, Eun-Joon Chun

**Affiliations:** Department of Materials System Engineering, Pukyong National University, Busan 48513, Republic of Korea; tjdwls2@pukyong.ac.kr

**Keywords:** gas turbine blade, welding, 247LC superalloy, Hf-free filler metal, hot cracking temperature range, square-groove welding

## Abstract

In this study, based on previous fundamental research on weldability, we ultimately aim to propose a filler metal that enables hot crack-free repair welding of 247LC superalloy while minimizing compositional modification. First, we investigated the liquation cracking susceptibility of two candidate filler metals, namely Hf-free and B-free 247LC superalloy welds, by individually removing Hf and B and performing a spot-Varestraint test. As a result, the liquation cracking temperature range (LCTR) of B-free 247LC was 370 K and 230 K for Hf-free 247LC. The results indicated a significant reduction in the liquation cracking temperature range (LCTR) to 230 K for the Hf-free alloy, from 620 K for the Hf-containing standard 247LC alloy. Direct microstructural analysis of the liquation cracking surfaces revealed a higher liquation initiation temperature at the γ/MC interface in the Hf-free alloy, ranging from 1460 to 1600 K, compared to that of the original 247LC alloy composition, which contributed to the reduced LCTR. These findings indicate that Hf-free 247LC superalloys offer enhanced weldability—particularly for manufacturing and repairing critical components of tools with high-temperature applications, such as gas-turbine blades. Finally, assuming the Hf-free 247LC alloy as a filler metal and the original 247LC alloy composition as a base metal, double square groove welding was performed. This clearly confirmed the possibility of hot crack-free welding with Hf-free 247LC filler metal, effectively suppressing both liquation and solidification cracking simultaneously.

## 1. Introduction

The directionally solidified 247LC superalloy is a key material for high-temperature components, such as gas-turbine blades and vanes [1,2,3]. During the manufacturing and repair of these components, welding is often applied. However, the 247LC alloy has poor weldability and is highly susceptible to weld hot cracking, including solidification and liquation cracking during the welding process [4,5,6,7]. Therefore, the development of tailored filler metals is an essential technology for the 247LC superalloy. However, particularly for the 247LC superalloy, fundamental investigations into its hot cracking susceptibility are extremely insufficient. In this regard, our research group has extensively evaluated the susceptibility of the 247LC superalloy and investigated potential filler metals that suppress both solidification and liquation cracking. At the same time, we have examined the welding metallurgical mechanisms underlying each type of cracking behavior. The following is a brief review of our findings regarding the hot cracking susceptibilities of 247LC superalloy welds.

Kim et al. were the first to evaluate the solidification cracking susceptibility of 247LC superalloy weld metal using a transverse Varestraint cracking test with gas tungsten arc welding (GTAW). They reported a wide solidification brittle temperature range (BTR) of 400 K for the 247LC alloy [8]. In other words, they explained that the severe weld solidification cracking behavior was attributed to the alloy’s wide BTR of 400 K, which was regarded as a wide solid–liquid coexistence temperature range during weld solidification. Also, the BTR of the alloy was reported to be reduced to 275 K when arc oscillation was applied without changing the weld metal composition [8] and further reduced to 217 K when a commercial filler metal was used [9], thereby reducing the solid–liquid coexistence temperature range during weld solidification due to its inherently narrow mushy zone range. In the same context, solidification crack-free welding metals could be achieved using ERNiCrMo-3, which is a similarly weldable alloy to ERNiCrMo-3 [9]. However, the use of weldable commercial fillers is expected to result in significant degradation of high temperature mechanical properties compared to the base metal, primarily due to the substantial differences in chemical composition between the filler and the base metal. In this regard, the use of B- and Hf-free 247LC filler metals was investigated, as these elements were regarded as harmful from the viewpoint of solidification cracking susceptibility based on previous studies [8], widening the solid–liquid coexistence temperature range and easily segregating during weld solidification. As a result, the Hf-free alloy demonstrated a remarkable reduction in the BTR from 400 K to 172 K, compared to the BTR reduction in the B-free alloy [10]. The identified mechanism for the reduced BTR in the Hf-free 247LC alloy was reported to be the suppression of MC carbide formation, which typically occurs at the terminal stage of weld solidification, thereby reducing the weld mushy zone range [10]. Namely, the Hf-free superalloy could be regarded as a potential filler metal in terms of solidification cracking susceptibility. However, the potential filler should also be investigated from the viewpoint of liquation cracking susceptibility in the partially melted zone (PMZ), which is another serious cracking issue in the 247LC and similar alloys [11,12,13,14]. Furthermore, as a final step, the weld validation results must support whether hot crack-free welds can be achieved when Hf-free 247LC is actually applied as a filler metal in real welding processes. Furthermore, the applicability of Hf-free 247LC filler for localized repair welding of the edge-missing part of gas turbine blades will be discussed.

From this perspective, it is essential to review previous studies on the hot cracking behavior of additively manufactured 247LC superalloys, which are highly regarded for repair applications, and to clarify their limitations. Adegoke et al. investigated the soundness of additively manufactured 247LC superalloy without modifying its chemical composition [15]. Their findings confirmed that solidification cracking occurred severely under all printing conditions. Carter et al. also used the original composition of the 247LC alloy and explored a newly designed printing pattern, namely the island scan strategy, which promotes a partially directionally solidified microstructure along with an extremely fine microstructure simultaneously [16]. However, they could not suppress hot cracking and were unable to establish a clear correlation between their new pattern strategy and hot cracking behavior. Gerstgrasser et al. also investigated hot crack-free 247LC alloy using focal shift during printing [17]. They suggested that the superior density and crack-free printed sample were achieved due to remelting and crack healing phenomena by utilizing optimal beam shifting during printing. However, using focal shift for printing does not inherently suppress hot cracking, nor does it guarantee that all cracks will be completely eliminated through remelting-induced crack healing. These limitations must be considered. In addition, several previous studies have addressed the hot crack formation behavior of 247LC alloy during additive manufacturing [18]. However, most of them are limited by the fact that they do not deviate from the original chemical composition. On the other hand, Griffiths et al. explored the modification and optimization of 247LC alloy composition for crack-free additive manufacturing [19]. They also recommended Hf-free 247LC to reduce hot cracking sensitivity and suggested that the mechanism behind the reduced hot cracking sensitivity is that the non-addition of Hf significantly reduces the solid–liquid coexistence temperature range during printing [20,21]. This result was well correlated with the BTR results of the Hf-free 247LC alloy, as evaluated by the Varestraint test [8]. However, they could not distinguish the effect of the non-addition of Hf on solidification and liquation cracking sensitivity during printing.

In this study, based on our previous research on solidification cracking susceptibility [8], liquation cracking susceptibility—specifically, the liquation cracking temperature range (LCTR)—was additionally evaluated for Hf- and B-free 247LC superalloy, which was considered a potential filler metal for assessing the repair welding of a damaged gas turbine blade tip. Moreover, the liquation cracking susceptibilities were compared with those of the original composition of 247LC alloys. The differences in liquation cracking susceptibilities were discussed based on microstructural analysis and thermodynamic calculations. Finally, based on the results, the optimal potential filler for the 247LC alloy was proposed and initially validated through real welding trials.

## 2. Materials and Methods

### 2.1. Hf- and B-Free 247LC Superalloys

Table 1 presents the chemical composition of the Hf- and B-free 247LC superalloy used in this study, along with the original composition of the 247LC superalloy. The Hf- and B-free 247LC alloys, including the original composition, were cast into plates with dimensions of 150 mm × 50 mm × 3 mm, and the as-cast materials were used without any post-casting heat treatment. To specifically assess the effect of non-addition of Hf on the susceptibility to liquation cracking, no post-casting heat treatment was applied in this study.

### 2.2. Spot-Varestraint Weld Cracking Test

**Figure 1:**
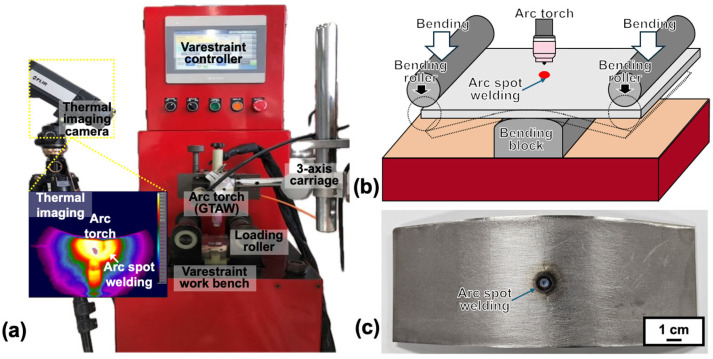
(**a**) Appearance and (**b**) schematic of the Spot-Varestraint test setup, and (**c**) appearance of the sample after the test.

The susceptibility of the Hf- and B-free 247LC superalloy to liquation cracking was evaluated by the Spot-Varestraint test with spot GTAW. Figure 1 illustrates the (a) testing machine and (b) setup. The central portion of each specimen was subjected to spot GTAW for 10 s, followed by the application of bending strain to induce forced high-temperature cracking in the weld zone. Figure 1c displays the sample after the Varestraint test. Table 2 presents the test conditions. The bending strain applied was 2.5%, and the deformation rate was 400 mm/s. ending strain was determined using the following equation [22,23,24]:

(1) $$\varepsilon =\frac{t}{2R}\times 100$$

where *R* is the radius of the testing specimen, *t* is the thickness of the specimen.

To confirm the reliability of the test results, each material was tested three times. A thermal imaging camera (A655sc FLIR, FLIR Systems, Wilsonvile, OR, USA) was used to monitor the temperature of the cracking site in real time, and these values were used to determine the temperature range over which cracking occurred. The test conditions used in this study were consistent with those employed in prior studies on the liquation cracking susceptibility of 247LC superalloy welds [11].

### 2.3. Double-Square-Groove Welding

**Figure 2:**
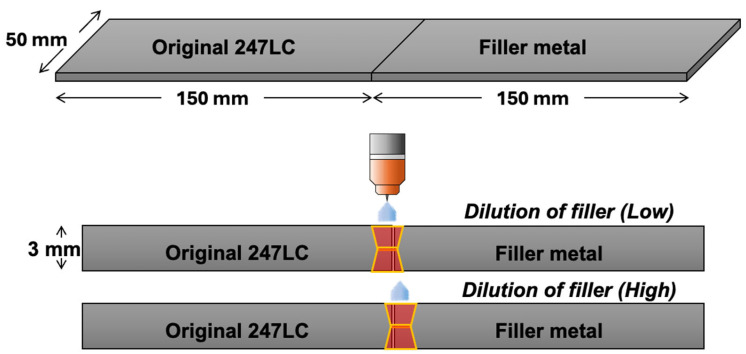
Schematic representation of double square groove welding specimens and arrangements.

For the validation of evaluated liquation cracking susceptibilities of potential filler metals, namely Hf- or B-free 247LC alloys, double-square-groove GTAW was performed, with one side of the welding plate serving as the filler metal. For specimens of the same size as those used in the Varestraint test, square-groove welding was carried out on both the upper and lower sides of the plates, as shown in Figure 2. To compare the dilution effect of the filler metal, the dilution was controlled under two conditions: lower and higher dilution of fillers. The welding conditions were as follows: an arc voltage of 10 V, an arc current of 60 A, a welding speed of 1 mm/s, and the use of Ar (purity 99.99%) as the shielding gas.

### 2.4. Microstructural Analysis

The microstructure of the hot cracks produced in the Varestraint test was analyzed. The fracture surfaces were examined via scanning electron microscopy (SEM; SU5000, Hitachi Ltd., Tokyo, Japan). The elemental distribution of the cracks was determined using electron probe microanalysis (EPMA; JXA-8530F, JEOL Ltd., Tokyo, Japan). To accurately identify the phases contributing to the cracking, transmission electron microscopy (TEM; JEM-F200, JEOL Ltd., Japan) was conducted. Elemental analysis by energy-dispersive X-ray spectroscopy (EDS; JED-2300, JEOL Ltd., Japan) was performed during the TEM analysis. The test samples for TEM were prepared using a focused ion beam (FIB; Crossbeam 550, ZEISS, Baden-Württemberg, Germany). The microstructure and the presence of hot cracks in double square groove welds were analyzed using an optical microscope (OM; Leica DM IRM, Leica Microsystems, Hessen, Germany).

## 3. Results and Discussion

### 3.1. Liquation Cracking Behavior of Hf- and B-Free 247LC Superalloy Welds

**Figure 3:**
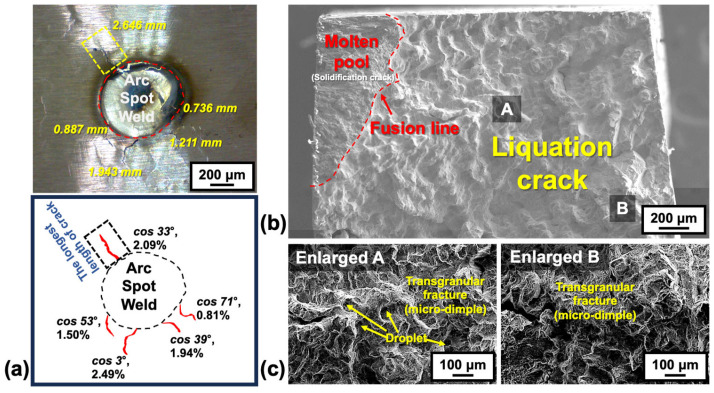
Results of the spot-Varestraint test for Hf-free 247LC: (**a**) photograph and schematic of liquation cracking behavior after the spot-Varestraint test, and typical liquation cracking surfaces at (**b**) low and (**c**) high magnifications.

**Figure 4:**
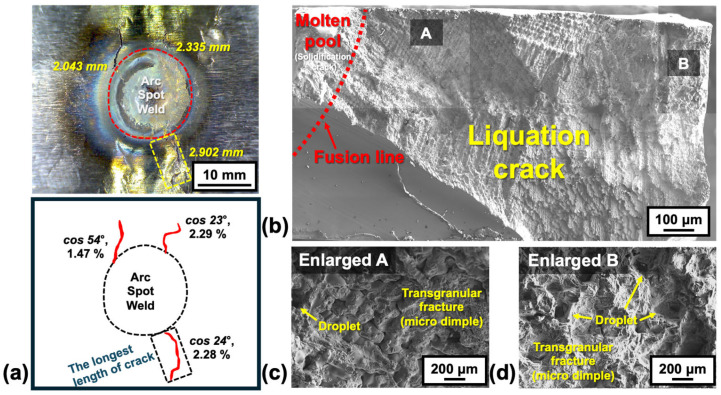
Results of the spot-Varestraint test for B-free 247LC: (**a**) photograph and schematic of liquation cracking behavior after the spot-Varestraint test, and typical liquation cracking surfaces at (**b**) low and (**c**,**d**) high magnifications.

Figure 3 and Figure 4 show representative results of the spot-Varestraint test for Hf-free and B-free 247LC, respectively. In particular, Figure 3a and Figure 4a show the surface appearance after the spot-Varestraint test for the Hf-free and B-free 247LC alloys. For both cases, a significant amount of hot cracking was observed in the heat-affected zone (HAZ), specifically outside the fusion line. Figure 3b and Figure 4b show an SEM image of the longest crack surface from the test. Enlarged views are also presented in Figure 3c and Figure 4c for both materials. All the fracture surfaces exhibited a combination of intergranular fracture and fine dimples, which are characteristic features of liquation cracking [25]. This indicates that the hot cracks produced in the spot-Varestraint test were all liquation cracks in the PMZ for both Hf-free and B-free 247LC alloys.

### 3.2. Effect of Hf and B Removal on LCTR

**Figure 5:**
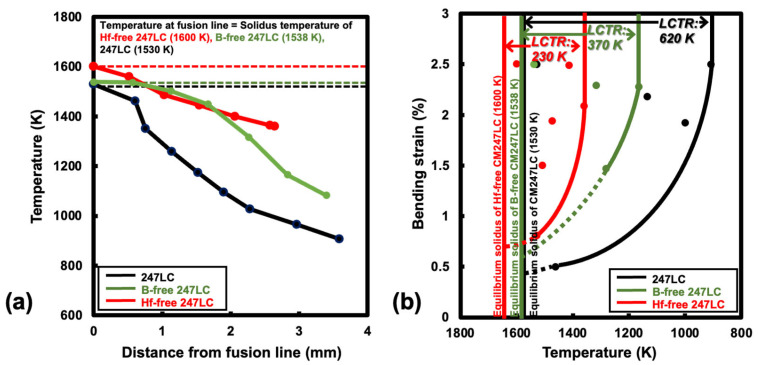
Determination of the LCTR for the Hf- and B-free 247LC superalloy welds: (**a**) temperature profile at the liquation cracking site during the Spot-Varestraint test; (**b**) high-temperature ductility curve for the liquation cracking.

Figure 5 presents the evaluation results for the LCTR of Hf-free and B-free 247LC superalloys. These results were compared with the previously reported LCTR of the original 247LC alloy composition [11]. Figure 5a shows the temperature profiles at the liquation cracking site during the Varestraint test, as measured by using the thermal imaging camera. Using the temperature data, we constructed the high-temperature ductility curve for the liquation cracking, as shown in Figure 5b. The strain values used in the ductility curve were calculated by correcting the applied bending strain (2.5%) for the crack initiation angle (i.e., cosθ). The effective strain values (cosθ) used in constructing Figure 5b are indicated in the schematic of the cracking behavior shown in Figure 3a and Figure 4a. The LCTR of the B-free 247LC alloy was determined to be 370 K, while that of the Hf-free 247LC alloy was 230 K, with saturation occurring at an effective strain of approximately 2%. In comparison, the original as-cast 247LC alloy had an LCTR of 620 K [11]. Therefore, we first confirmed that the individual absence of B and Hf in the 247LC alloy had a considerable impact on reducing the LCTR by 250 K and 390 K, respectively.

### 3.3. Welding Metallurgical Mechanism of Reduced LCTR in Hf-Free 247LC Superalloy Welds

From the previous results displayed in Figure 5, the Hf-free 247LC alloy showed a particularly significant reduction in LCTR compared to the B-free 247LC alloy. In other words, the alloy could be sufficiently regarded as a potential filler metal for the 247LC superalloy. Consequently, the welding metallurgical mechanism behind the LCTR reduction is discussed mainly for the Hf-free 247LC in this section. In previous studies, liquation cracking in the welds of 247LC superalloys was attributed to constitutional liquation at the γ/carbide interface, occurring considerably below the equilibrium solidus temperature of the alloy during the heating stage of the welding process [11]. Therefore, the reduction in LCTR in the Hf-free 247LC alloy was also discussed based on the constitutional liquation behavior at the γ/carbide interface by directly analyzing the liquation cracking surface.

As reported in detail by previous studies, the LCTR evaluated in the Varestraint test is believed to be influenced by the temperature difference between the compositional liquation temperature of the γ/carbide interface and the equilibrium solidus temperature of the alloy [11]. In other words, liquation cracking in the PMZ occurs when localized liquation and separation of the liquid film take place at compositional liquation temperatures lower than the equilibrium solidus temperature, leading to liquation cracking. Thus, the expansion or contraction of the LCTR can be understood as a result of changes in the local liquation initiation temperature, either increasing or decreasing.

**Figure 6:**
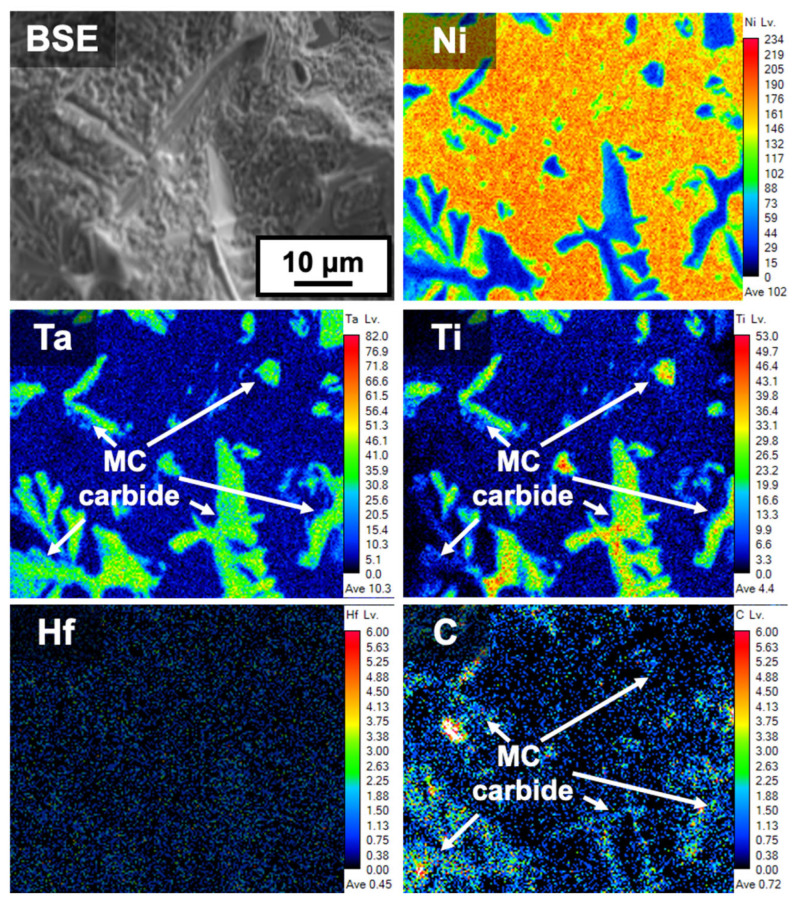
Microstructure and elemental distribution (Ni, Ta, Ti, Hf, C) of the liquation cracking surface for Hf-free 247LC superalloy welds.

**Figure 7:**
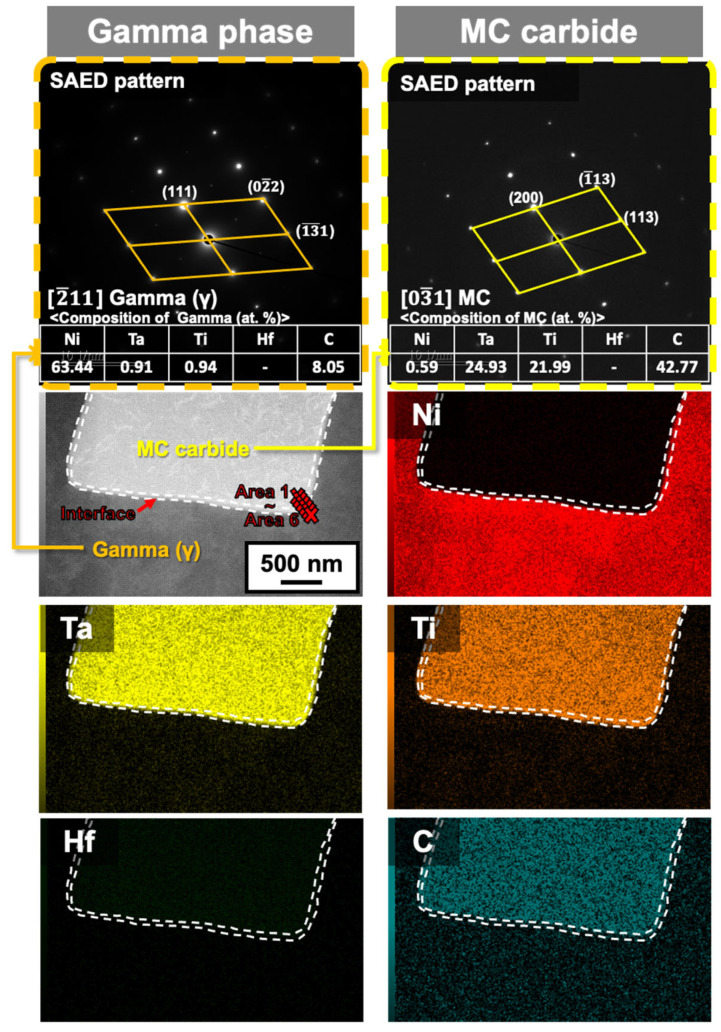
Representative images of the interaction layer between MC carbide and the matrix gamma phase for the Hf-free 247LC specimen analyzed via TEM and EDS.

Figure 6 shows the EPMA results for the liquation cracking surface shown in Figure 3b. Based on the Ni matrix element, the presence of Ti- and Ta-rich carbides was clearly confirmed. Notably, the absence of the Hf element was attributed to the design of the Hf-free 247LC alloy. Similar results were observed in other liquation cracking regions. To investigate the constitutional liquation behavior, TEM analysis was directly performed on the liquation cracking surface in the same region as Figure 6. Figure 7 presents representative results of the TEM analysis. The TEM results revealed the presence of MC carbides along with the γ matrix. Six areas (from Area #1 to Area #6, step size: 2 nm) along the γ/MC interface were analyzed to determine the local chemical composition for the estimation of the liquation initiation temperatures. Table 3 presents the compositional analysis results for each area at the interface. Compared to the results obtained from the original composition of the 247LC alloy, Hf was not detected in the Hf-free 247LC alloy [9]. Based on the compositional analysis results, the solidus temperatures (estimated as the liquation initiation temperature) at each interface region were calculated using Thermo-Calc software (2025a, database: TCNi12).

**Figure 8:**
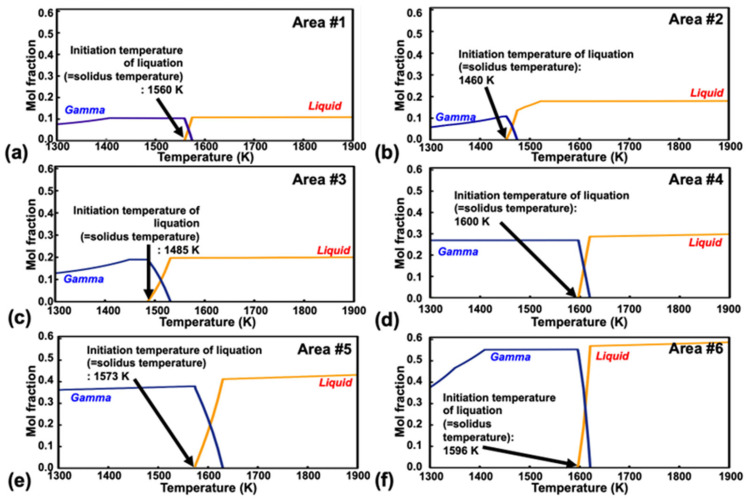
Calculation of the local solidus temperature (i.e., liquation initiation temperature) at the γ/MC interface of Hf-free 247LC using Thermo-Calc software: (**a**) Area #1, (**b**) Area #2, (**c**) Area #3, (**d**) Area #4, (**e**) Area #5, and (**f**) Area #6.

As displayed in Figure 8, the local liquation temperature range for the γ/MC interface in the Hf-free 247LC superalloy welds was calculated to be 1460–1600 K. In contrast, the liquation temperature range at the γ/MC interface of the original 247LC alloy was reported to be 1125–1356 K [11]. This indicates that the liquation temperature of the Hf-free alloy is approximately 335 K higher than that of the original 247LC alloy. Namely, the difference between the equilibrium solidus and the liquation initiation temperature was considerably reduced, supporting the decreased liquation cracking susceptibility and the 390 K reduction in LCTR observed in this study. As reported, due to its low partition coefficient, Hf tends to easily segregate at grain boundaries, forming a low-temperature liquid during cast solidification. These regions can then easily undergo liquation when exposed to welding heat effects [26,27]. Therefore, the increased liquation initiation temperature of the Hf-free 247LC alloy, compared to that of the Hf-containing 247LC alloy, can be attributed to the absence of Hf, which increases the local melting point at the grain boundaries. This is considered to be a key factor contributing to the reduction in LCTR in Hf-free 247LC.

### 3.4. Weldingvalidation of Hf-Free 247LC Filler Metal Hot Crack-Free 247LC Superalloy Welds

**Figure 9:**
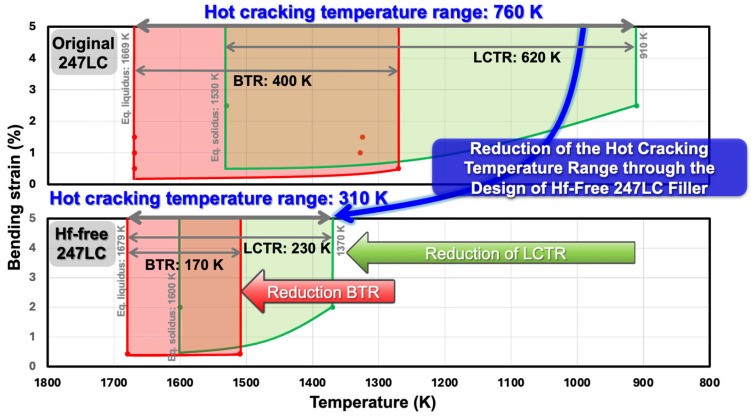
Summarization of BTR and LCTR for original composition of 247LC and Hf-free 247LC alloys.

**Figure 10:**
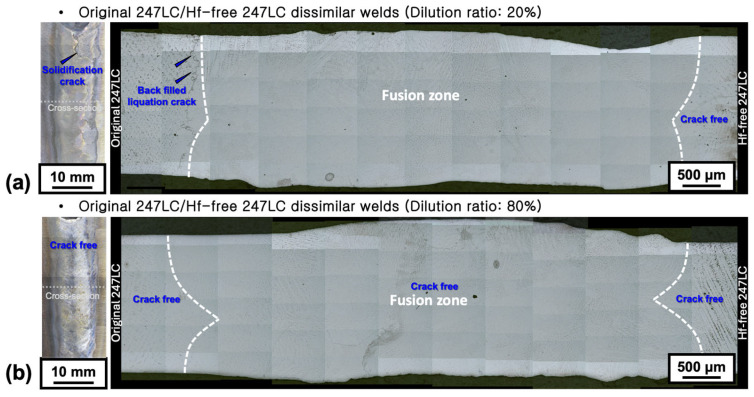
Cross-sectional macrostructure of double square groove welds assuming Hf-free 247LC as a filler metal: (**a**) 20% and (**b**) 80% dilution ratio of Hf-free 247LC.

Figure 9 presents a comprehensive comparison between the previous study results on the reduction in BTR in Hf-free 247LC alloy [10] and the LCTR reduction results of Hf-free 247LC alloy confirmed in this study, in relation to the original 247LC alloy. Even when the overlapping temperature range between BTR and LCTR is excluded, the hot cracking temperature range of the original 247LC alloy is 760 K, whereas that of the Hf-free 247LC alloy is reduced to approximately half, measuring 310 K, solely due to the absence of Hf. Therefore, the Hf-free 247LC alloy is considered highly promising as a filler metal for localized gas turbine blade repair welding from the perspective of the hot cracking temperature range. Accordingly, the possibility of crack-free welding was examined through actual welding validation. Figure 10 presents the results of double square groove welding using Hf-free 247LC alloy as a filler metal. In both the surface and cross-sectional images, the left-side material relative to the weld is the original 247LC alloy, while the right-side material is Hf-free 247LC. As observed in Figure 10a, when the dilution ratio of the filler metal (Hf-free 247LC) is 20%, solidification cracking is present. However, when the dilution ratio increases to 80% (Figure 10b), no solidification cracks are observed in any surface or cross-sectional regions. Furthermore, in the case of PMZ liquation cracks, no cracks were found on the PMZ of the Hf-free 247LC alloy, regardless of the dilution ratio. Therefore, due to the reduced hot cracking temperature range of the Hf-free 247LC alloy, it has been verified that solidification and liquation cracking are effectively suppressed even during actual welding processes.

## 4. Conclusions

The objective of this study was to evaluate the feasibility of applying the Hf-free 247LC superalloy as a potential filler metal to achieve hot crack-free 247LC superalloy welds for the successful repair welding of high-temperature components in gas turbine engines. Based on the author’s previous studies on the solidification cracking susceptibility of Hf-free 247LC alloy, the liquation cracking susceptibility in the PMZ was quantitatively evaluated. Additionally, the applicability of Hf-free 247LC as a potential filler metal was validated through actual welding trials:

- The as-cast Hf-free 247LC alloy exhibited an LCTR of 230 K, which is approximately 390 K lower than that (620 K) of the original 247LC alloy. This reduction indicates that the Hf-free 247LC alloy has a higher resistance to liquation cracking than the original alloy.
- Microstructural analysis of the crack surfaces indicated that liquation cracking in the Hf-free 247LC alloy primarily occurred at the γ/MC interface. The liquation initiation temperature for the Hf-free alloy was between 1460 and 1600 K, which was significantly higher than the liquation initiation temperature range of 1125–1356 K observed for the original Hf-containing 247LC alloy. The higher liquation initiation temperature of the Hf-free alloy was correlated with its reduced LCTR and contributed to its lower liquation cracking susceptibility.
- Based on these results, the welding validation of Hf-free 247LC filler metal was conducted by performing double square groove welding with the original composition of the 247LC alloy. The Hf-free 247LC filler metal demonstrated excellent weldability in terms of both liquation and solidification cracking susceptibilities, effectively suppressing both types of hot cracking. Namely, these results make it a promising candidate for the manufacturing and repair of gas turbine blades and other critical components.

## Figures and Tables

**Table 1:** Chemical compositions of the materials used (mass%).

Materials	Ni	Cr	Co	Mo	W	Ta	Ti	Al	C	B	Zr	Hf	P	S
247LC	Bal.	8.1	9.2	0.5	9.5	3.2	0.7	5.6	0.07	0.015	0.015	1.4	<0.00006	<0.00002
B-free 247LC	Bal.	8.3	9.3	0.5	9.6	3.3	0.8	5.6	0.07	0	0.007	1.4	<0.00006	<0.00002
Hf-free 247LC	Bal.	8.3	9.3	0.5	9.6	3.3	0.8	5.6	0.07	0.015	0.007	0	<0.00006	<0.00002

**Table 2:** Conditions of the Spot-Varestraint test.

Parameter	Setting
Heat source	GTAW
Arc voltage (V)	18
Arc current (A)	60
Heat input (J/mm)	648
Arc length (mm)	2
Welding time (s)	10
Shielding gas	Ar
Bending rate (mm/s)	400
Bending strain (%)	2.5

**Table 3:** EDS results for the interface between gamma and MC carbide (mass%).

Analysis Points	Ni	Cr	Co	Mo	W	Ta	Ti	Al	C	B	Zr	Hf
Area #1	Bal.	1.68	1.21	0.34	0.84	64.61	16.96	0.21	8.11	-	0.46	-
Area #2	Bal.	2.08	1.98	0.15	1.52	61.59	16.93	0.17	7.06	-	0.04	-
Area #3	Bal.	2.69	2.18	0.82	0.84	58.49	16.47	0.25	7.3	-	0.86	-
Area #4	Bal.	3.74	3.71	0.32	3.29	49.88	14.64	0.47	7.11	-	0.29	-
Area #5	Bal.	5.32	4.71	0.89	3.98	40.08	11.98	0.9	6.43	-	0.59	-
Area #6	Bal.	6.71	5.65	1.6	6.02	30.1	10.23	2.2	4.38	-	0.2	-

## Data Availability

The original contributions presented in this study are included in the article. Further inquiries can be directed to the corresponding authors.
